# Metallothionein 1H (MT1H) functions as a tumor suppressor in hepatocellular carcinoma through regulating Wnt/β-catenin signaling pathway

**DOI:** 10.1186/s12885-017-3139-2

**Published:** 2017-02-28

**Authors:** Yulong Zheng, Lihua Jiang, Yongxian Hu, Cheng Xiao, Nong Xu, Jianying Zhou, Xinhui Zhou

**Affiliations:** 1 Department of Medical Oncology, The First Affiliated Hospital, School of Medicine, Zhejiang University, Hangzhou, 310003 China; 2 Department of Neurology, The Children’s Hospital, School of Medicine, Zhejiang University, Hangzhou, 31006 China; 3 Department of Hematology, The First Affiliated Hospital, School of Medicine, Zhejiang University, Hangzhou, 310003 China; 4 Department of Respiratory Diseases, The First Affiliated Hospital, School of Medicine, Zhejiang University, 79 Qingchun Road, Hangzhou, Zhejiang 310003 China; 5 Department of Gynecology, The First Affiliated Hospital, School of Medicine, Zhejiang University, 79 Qingchun Road, Hangzhou, Zhejiang 310003 China

**Keywords:** Metallothionein 1H (*MT1H*), Hepatocellular cancer (HCC), Proliferation, Invasion, Wnt/β-catenin

## Abstract

**Background:**

Metallothionein 1H (*MT1H*) expression level is downregulated in several kinds of tumors, including hepatocellular cancer (HCC). However, its biological functions and underlying mechanisms in HCC is largely unknown. The current study aimed to demonstrate the expression status, biological roles and potential mechanisms of MT1H in HCC.

**Methods:**

We investigated the expression level of *MT1H* in the Cancer Genome Atlas (TCGA) dataset and a panel of 12 paired tumor/non-tumor tissues. In vitro, gain-of-function experiments were performed to examine the role of MT1H on HCC cell proliferation, invasion, and migration. Using bioinformatics assay, reporter assays, quantitative real-time PCR, and western blotting, we explored the possible mechanisms underlying the role of MT1H in HCC cells. In vivo nude mice experiments were performed to assess the anti-proliferative role of MT1H in HCC.

**Results:**

Downregulation of *MT1H* was observed in TCGA dataset and a panel of 12 paired tumor/non-tumor tissues. Ectopic overexpression of *MT1H* in HepG2 and Hep3B cells inhibited cell proliferation, invasion, and migration. Gene Set Enrichment Analysis (GSEA) showed that MT1H might involve in regulation of Wnt/β-catenin pathway. Top/Fop reporter assay confirmed that MT1H had an effect on Wnt/β-catenin signaling. Real-time PCR showed *MT1H* expression decreased the expression of Wnt/β-catenin target genes. Western blotting assay showed that overexpression of *MT1H* inhibited the nuclear translocation of β-catenin and that the Akt/GSK-3β axis mediated the modulatory role of MT1H on Wnt/β-catenin signaling in HCC. In vivo nude mice experiments demonstrated that MT1H suppressed the proliferation of HCC cells. Taken together, MT1H suppressed the proliferation, invasion and migration of HCC cells via regulating Wnt/β-catenin signaling pathway.

**Conclusions:**

This study demonstrated that through inhibiting Wnt/β-catenin pathway, MT1H suppresses the proliferation and invasion of HCC cells. *MT1H* may be a potential target for HCC therapy.

**Electronic supplementary material:**

The online version of this article (doi:10.1186/s12885-017-3139-2) contains supplementary material, which is available to authorized users.

## Background

Hepatocellular cancer (HCC), the third cause of cancer-related death worldwide, accounts for 85%-90% of all primary liver cancer [1, 2]. In China, it is the most commonly diagnosed cancer and the leading cause of cancer death in male patients younger than 60 years old [3]. Although there are various treatment options, such as surgical resection, liver transplantation, interventional treatment, and systemic therapies, the outcomes of patients with advanced stage are still extremely poor [4, 5]. Conventional cytotoxic systemic therapy is of limited benefit for unresectable or metastatic HCC [6]. Sorafenib, a molecular-targeted agent, demonstrates a modest overall survival benefit, however resistance develops eventually [6–8]. Deeper understanding of molecular carcinogenesis of HCC is urgently warranted to develop new novel target agents.

The metallothioneins (MTs) are a class of cysteine-rich, low-molecular-weight, metal-binding intracellular proteins, including four identified isoforms (MT-1, MT-2, MT-3, and MT-4) [9, 10]. In human, these proteins are encoded by a cluster of genes, which are located on chromosome 16q13 [11]. The MT-1 protein comprises many subtypes encoded by a set of *MT-1* genes (*MT1A, MT1B, MT1E, MT1F, MT1G, MT1H*, etc.). *MT-1* genes are reported to be involved in carcinogenesis in various human tumors [9]. In Fu et al’s study [12], MT1G acts as a tumor suppressor in thyroid carcinogenesis via regulating the phosphatidylinositol-3-kinase (PI3K)/Akt pathway and Rb/E2F pathway. Loss of heterozygosity (LOH) causes the downregulation of *MT1F* in colon cancer tissues, suggesting a tumor suppressor role for MT1F in colon cancer [13]. Of specific note, by analyzing 30 sets of online microarray data, Han et al. [14] found a consistent downregulation of *MT1H* in various kinds of human malignancies as compared with normal tissues, including small cell lung cancer, neuroblastoma, melanoma, B-cell lymphoma, prostate cancer, colon cancer, breast cancer, and leukemia. Furthermore, a 10- to 100-fold decrease of *MT1H* expression was observed in HCC in comparison with normal liver tissues, indicating a potential role of MT1H in the development and progression of HCC [14]. Nevertheless, the biological functions and underlying mechanisms of MT1H in HCC are largely unknown.

The Wnt/β-catenin signaling pathway is frequently activated during carcinogenesis, especially in HCC [15]. In the canonical Wnt pathway, Wnt binding to Fz receptor inactivates the β-catenin destruction complex of adenomatous polyposis coli (APC), axin, and glycogen synthase kinase-3 β (GSK-3β) [15]. When the Wnt pathway is activated, β-catenin is released from the complex and translocated into nucleus. The nuclear β-catenin binds to members of the lymphoid-enhancing factor/T-cell factors (LEF/TCF) family that activate target genes transcription [16]. Further delineation of the mechanisms underlying the dysregulated Wnt/β-catenin signaling in HCC is of great interest.

In the current study, we identified the biological functions of MT1H in HCC and explored the possible mechanisms. Our study suggests that MT1H plays crucial role in regulating the proliferation and invasion of HCC cells through modulating Wnt/β-catenin signaling.

## Methods

### Cells and culture

Human hepatoblastoma cell lines HepG2 and Hep3B were obtained from the China Infrastructure of Cell Line Resource. The cells were cultured in Dulbecco’s modified Eagles medium (DMEM) (Gibco, Grand Island, NY) containing 10% fetal bovine serum (FBS), 100 U/mL penicillin, and 100 mg/mL streptomycin (Gibco, Grand Island, NY) at 37 °C in a humidified atmosphere with 5% CO_2_.

### Obtainment of clinical specimens

Twelve HCC tissues (T) and their corresponding adjacent non-tumorous liver tissues (NT) were obtained from the surgery operation in the First Affiliated Hospital of Zhejiang University from Jan. 2015 to Dec. 2015. NT was defined as liver tissues more than 2 cm away from the edge of the tumor [17]. Two pathologists carried out histopathological diagnosis of the specimens independently. Soon after the tissues were collected, they were immediately snap-frozen in liquid nitrogen and stored at −80 °C for subsequent total cellular RNA extraction. The clinicopathologic characteristics of the patients are listed in Table 1. This study was performed in accordance with the ethical guidelines of the *Declaration of Helsinki* and was approved by the hospital’s Institutional Review Board (No. 2016397). Informed consent was obtained from each patient.

**Table 1:** The characteristics of patients (*n* = 12)

Clinical characteristics		Patients (*n*, %)
Age (yr)	≤50	4 (33.3)
	>50	8 (66.7)
Gender	Male	9 (75)
	Female	3 (25)
BCLC stage	Stage 0-A	3 (25)
	Stage B	7 (58.3)
	Stage C-D	2 (16.7)
Etiology	Alcoholic	3 (25)
	HBV	6 (50)
	HCV	1 (8.3)
	Others	2 (16.7)
Liver cirrhosis	No	2 (16.7)
	Yes	10 (83.3)

### Generation of stable cell lines with *MT1H* overexpression

Human TrueORF Gold™ pCMV6-Entry-MT1H plasmid with a C-terminal fusion of MYC/DDK tag was purchased from OriGene Technologies (Rockville, MD). To establish stable cell lines with constitutive expression of MT1H, HepG2 and Hep3B cells were transfected with pCMV6-Entry-MT1H by Lipofectamin™ 2000 (Invitrogen, Life Technology, Carlsbad, CA) according to the manufacturer’s protocol. After selection with complete medium containing G418 (0.6 mg/mL) for 2 weeks [18], individual clones were isolated and grown separately in the presence of G418. The expression of MT1H was confirmed by Western blotting assay. A stable transfectant expressing pCMV6-Entry empty vector was established and served as the control.

### Transwell invasion and migration assays

For Transwell invasion/migration assays, the indicated 2 × 10^4^ cells suspended in 200 μL DMEM without FBS were added to Transwell insert (Millipore, Billerica, MA) with or without coated Matrigel (BD Biosciences San Diego, CA), respectively. The insert held in 24-well companion plates with DMEM containing 10% FBS. After 24 h incubation, the cells and Matrigel in the upper chambers were removed by cotton tip. Migrating and invading cells at the bottom of the filter were fixed, stained with 1.0% crystal violet solution and photographed under a light microscope. The number of migrating and invading cells was quantified by counting five fields in each chamber [19]. This experiment was repeated three separate times.

### Wound healing assay

To evaluate the cell motility, the wound-healing assay was performed. Indicated cells were seeded in 6-well plates and grown into a monolayer. In order to exclude the possible role of MT1H in proliferation that may affect the result of wound healing assay, the cells were cultured in DMEM medium supplemented with lower percentage of serum (3%). A sterile p200 pipette tip was used to make a scratch and the cells were rinsed several times with medium to remove debris and unattached cells. Cells were then cultured for 36 h and the motility of cells was photographed. Migratory activity of the indicated cells was assessed by the width of the wound, and results were quantified as the percent of control [20].

### MTT (3-[4, 5-dimethylthiazol-2-yl]-2, 5-diphenyl-tetrazolium bromide) assay

HepG2-Vector, HepG2-MT1H, Hep3B-Vector and Hep3B-MT1H cells were trypsinized and seeded in 96-well plates at a density of 2 × 10^3^ cells/well. A solution of MTT (Sigma-Aldrich, St Louis, MO, 25 μL of a 5 mg/mL solution in PBS) was added and cells were incubated for another 2 h at 37 °C. Then the medium was replaced by 100 μL Dimethyl sulfoxide (DMSO, Sigma-Aldrich, St Louis, MO) and shaken at room temperature for 10 min. The plate absorbance was measured at 490 nm. This experiment was repeated three separate times.

### Colony formation assay

Two hundred indicated cells per well were put in 12-well plates and cultured in complete medium for 10 days. The colonies were fixed with 4% paraformaldehyde and stained with 1% crystal violet solution [21]. This experiment was repeated three separate times.

### 5-Ethynyl-2′-deoxyuridine (EdU) incorporation assay

EdU incorporation assay was carried out using the Cell-Light™ EdU In Vitro kit (RiboBio, Guangzhou, China) according to the manufacturer’s instructions. Briefly, the indicated cells added with EdU (50 μM) were cultured for 2 h, and then fixed in 4% paraformaldehyde at room temperature for 30 min. TritonX (0.5%) was used to permeabilize the cells. After incubated with 1 × Apollo reaction mixture for 30 min, the cells were subsequently stained with Hoechst 33342 for 30 min at room temperature [22]. EdU positive cells were calculated with Zeiss fluorescent microscope system (Carl Zeiss, Thornwood, NY). This experiment was repeated three separate times.

### Western blotting assay

Briefly, the indicated cells were washed with PBS and lysed using Radioimmunoprecipitation assay buffer (RIPA) (150 mM NaCl, 1% NP-40, 0.5% Sodium deoxycholate, 0.1% SDS, 50 mM Tris–HCl pH 7.4) and the amounts of protein were assayed using the Bradford method. Equal amount of protein was subjected to sodium dodecyl sulfate-polyacrylamide gels electrophoresis (SDS-PAGE). Proteins on the gel were transferred onto a polyvinylidene difluoride (PVDF) membrane (Millipore, MA). The membrane was blocked in 5% powdered non-fat milk in Tris-buffered saline (TBS) solution containing 0.05% Tween 20 for 1 h. Membrane was then incubated with primary antibody overnight at 4 °C, followed by incubation with secondary antibody conjugated to horseradish peroxidase at room temperature for 1 h. The signal was developed using the enhanced chemiluminescence (ECL, Advance Western blotting detection kit, Pierce, Rockford, IL) to expose an Xray film. The primary antibodies against Flag, β-catenin, lamin a, α-Tubulin (Abcam, Cambridge, UK), phospho-Akt (Ser473), Akt, phospho-GSK-3β (Ser9), and GSK-3β (Cell Signaling Technology, Beverly, MA), were used. The experiment was repeated three times to confirm the results. The intensity of the indicated bands was quantified using Quantity One software (Bio-Rad, Richmond, CA).

### Immunofluorescence

Indicated cells were grown on coverslip (Fisher Scientific, Pittsburgh, PA) on 24-well plate were fixed with 4% paraformaldehyde and permeabilized with 0.1% Triton-X in PBS. β-catenin were detected by incubation with specific anti-β-catenin antibody (Abcam, Cambridge, UK) overnight at 4 °C. A secondary antibody (donkey anti-rabbit rhodamine red) (Jackson Immunoresearch Laboratories, West Grove, PA) was used before staining the nuclei with 1 μg/mL DAPI (Sigma-Aldrich, St. Louis). Pictures were acquired by using a fluorescence microscope (Olympus, Tokyo, Japan).

### Quantitative real-time PCR (qPCR) analysis

Total RNA was extracted from the cells or tissues using Trizol reagent (Invitrogen, Life Technology, Carlsbad, CA), and reverse transcription was conducted as follows with random primers and Moloney murine leukemia virus reverse transcriptase (Promega, Madison, Wisconsin) in accordance with the manufacturer’s protocol. The original amount of the specific transcripts were measured by real-time PCR according to a standard protocol with a SYBR Green PCR kit (Roche Diagnostics, Indianapolis, IN) using the ABI Prism 7000 sequence detection system (Applied Biosystems, Foster City, CA). For each gene and sample, three independent assays were conducted and the comparative Ct method was performed to calculate the relative abundance of mRNA compared with that of the endogenous reference control β-actin. The primers used were listed in Table 2.

**Table 2:** Sequences of primers

Gene	Forward Primer (5′-3′)	Reverse primer (5′-3′)
*MYC*	TTTCGGGTAGTGGAAAACCA	CACCGAGTCGTAGTCGAGGT
*MMP7*	GAGCTACAGTGGGAACAGGC	GCATCTCCTTGAGTTTGGCT
*LEF1*	TGGATCTCTTTCTCCACCCA	CACTGTAAGTGATGAGGGGG
*ACTB*	GCCAACACAGTGCTGTCTGG	CTCAGGAGGAGCAATGATCTTG

### Nuclear protein extraction

Nuclear protein extraction was carried out using Active Motif Nuclear Extraction Kit (Active Motif, Carlsbad, CA) according to the manufacture’s protocol. Lamin A was used as nuclear fraction controls [23].

### Luciferase reporter assay

The Top/Fop flash plasmid system (Upstate, Lake Placid, NY) was used to evaluate the transcriptional activity of TCF. Topflash and Fopflash plasmids were co-transfected in the indicated cells using Lipofectamin™ 2000 (Invitrogen, Life Technology, Carlsbad, CA). The luciferase values were measured after 48 h incubation, using Dual-Luciferase reporter assay system (Promega, Madison, WI) according to the manufacturer’s instructions [24]. The experiment was repeated three times to confirm the results.

### In vivo tumorigenicity assay

HepG2-Vector and HepG2-MT1H (1.5 × 10^6^ cells) were injected subcutaneously into the right flanks of female BALB/c athymic nude mice (4–6 weeks of age), five mice per group [25]. The tumor volume was measured routinely using a caliper every 5 days, and was calculated with the formula 0.5 × (length × width^2^). At 30 days after injection, all mice were sacrificed and tumors were weighted. This experiment was performed in accordance with the institutional ethical guidelines for animal experiment.

### Gene set enrichment analysis (GSEA)

GSEA (http://software.broadinstitute.org/gsea/index.jsp) was performed as previously described [26]. We used LIHC RNA-seq data generated by the Cancer Genome Atlas (TCGA) and sorted the samples into the top and bottom quartiles of MT1H expression (high and low MT1H expression, respectively). Analyzed gene sets (PID_WNT_CANONICAL_PATHWAY [27] and WNT_SIGNALING (http://software.broadinstitute.org/gsea/msigdb/cards/WNT_SIGNALING.html) were downloaded from GSEA and the genes are listed in Additional file 1: Table S1. To assess the enrichment, the absolute value of the GSEA metric (Pearson correlation) was considered for deregulated genes.

### Statistical analysis

All statistical analyses were performed using the SPSS 13.0 package (SPSS International, Chicago, IL, USA). Continuous variables were presented as mean ± SD and analyzed using the Student’s t-test. The relationship between *MT1H* expression and the expression of Wnt/β-catenin target genes was analyzed by Chi-square tests. *P* < 0.05 was considered statistically significant.

## Results

### *MT1H* expression in human HCCs

**Fig. 1:**
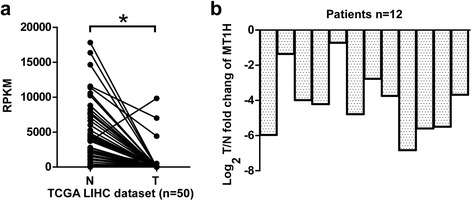
Low expression level of MT1H in HCC patients. **a** The expression of MT1H in 50 pairs of primary HCC tumors versus paired non-tumorous liver tissues mined from RNAseqV2 data set on TCGA. **b** The log_2_Tumor/Non-tumorous change of MT1H expression in 12 paired fresh HCC tissues and the adjacent non-cancerous liver tissues

To explore the role of MT1H in HCC, we analyzed the *MT1H* expression in large datasets derived from TCGA databases [26]. TCGA is a collaboration of the National Cancer Institute (NCI) and National Human Genome Research Institute (NHGRI) [26]. TCGA contains multi-dimensional and comprehensive maps of the key genomic changes in various types of cancer, including HCC. The results from TCGA RNASeq2 data showed that in 50 pairs of HCC tissues (T) and their corresponding adjacent non-tumorous liver tissues (NT), *MT1H* is significantly downregulated in most (48/50) HCCs (Fig. 1a). The clinical data of these 50 patients are shown in Additional file 2: Table S2. Furthermore, we expanded our investigation to clinical samples in order to assess the expression level of *MT1H* in 12 human HCCs by qRT-PCR. As shown in Fig. 1b, *MT1H* expression level was found to be markedly decreased in all HCC tumors as compared with the adjacent non-tumorous liver tissues. These results suggest that *MT1H* expression is downregulated in human HCCs and may play a role in hepatocellular carcinogenesis.

### MT1H suppresses the growth of HepG2 and Hep3B cells

**Fig. 2:**
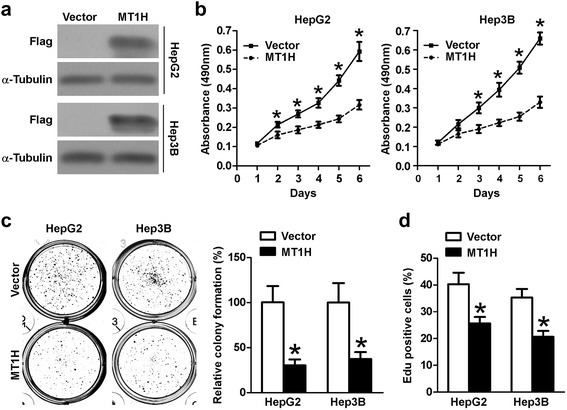
MT1H plays a pivotal role in HCC cell proliferation. **a** Expression of MT1H-Flag in indicated cells. Protein extracts from indicated cells were immunoblotted with antibody for Flag. α-Tubulin as a loading control. **b** HepG2-Vector, HepG2-MT1H, Hep3B-Vector, and HepG3B-MT1H cells was measured by MTT response. Error bars indicate standard deviation (Student’s *t*-test; *, *P* < 0.05). **c** Colony number of MT1H overexpressing HepG2 and Hep3B cells was significantly decreased as compared with respective vector-control. Error bars indicate standard deviation (Student’s *t*-test; *, *P* < 0.05). **d** Edu incorporation analysis indicates that MT1H suppresses the proliferation of HCC cells. Error bars indicate standard deviation (Student’s *t*-test; *, *P* < 0.05)

To examine the potential role of MT1H in hepatocellular carcinogenesis, we sought to assess the effect of MT1H on the growth of HCC cells. HepG2 and Hep3B cells were stably transfected with full length Flag-tagged MT1H and immunoblots were performed to confirm the overexpression of MT1H (Fig. 2a). As shown in Fig. 2b, ectopic overexpression of MT1H in HepG2 and Hep3B cells significantly inhibited the MTT response as compared with that of empty vector control cells. Next, we examined the effects of enforced expression of MT1H on the colony forming capability of HCC cells. As shown in Fig. 2c, enforced expression of MT1H in HepG2 and Hep3B cells decreased more than 50% in colony numbers as compared with the control cells. Moreover, in Edu incorporation assay, the DNA synthesis capability was significantly decreased in MT1H-overexpressed cells (Fig. 2d). The results of these experiments demonstrate that enforced expression of MT1H in HCC cells is correlated with impaired cell growth and DNA synthesis.

### MT1H suppresses tumor cell invasiveness and motility

**Fig. 3:**
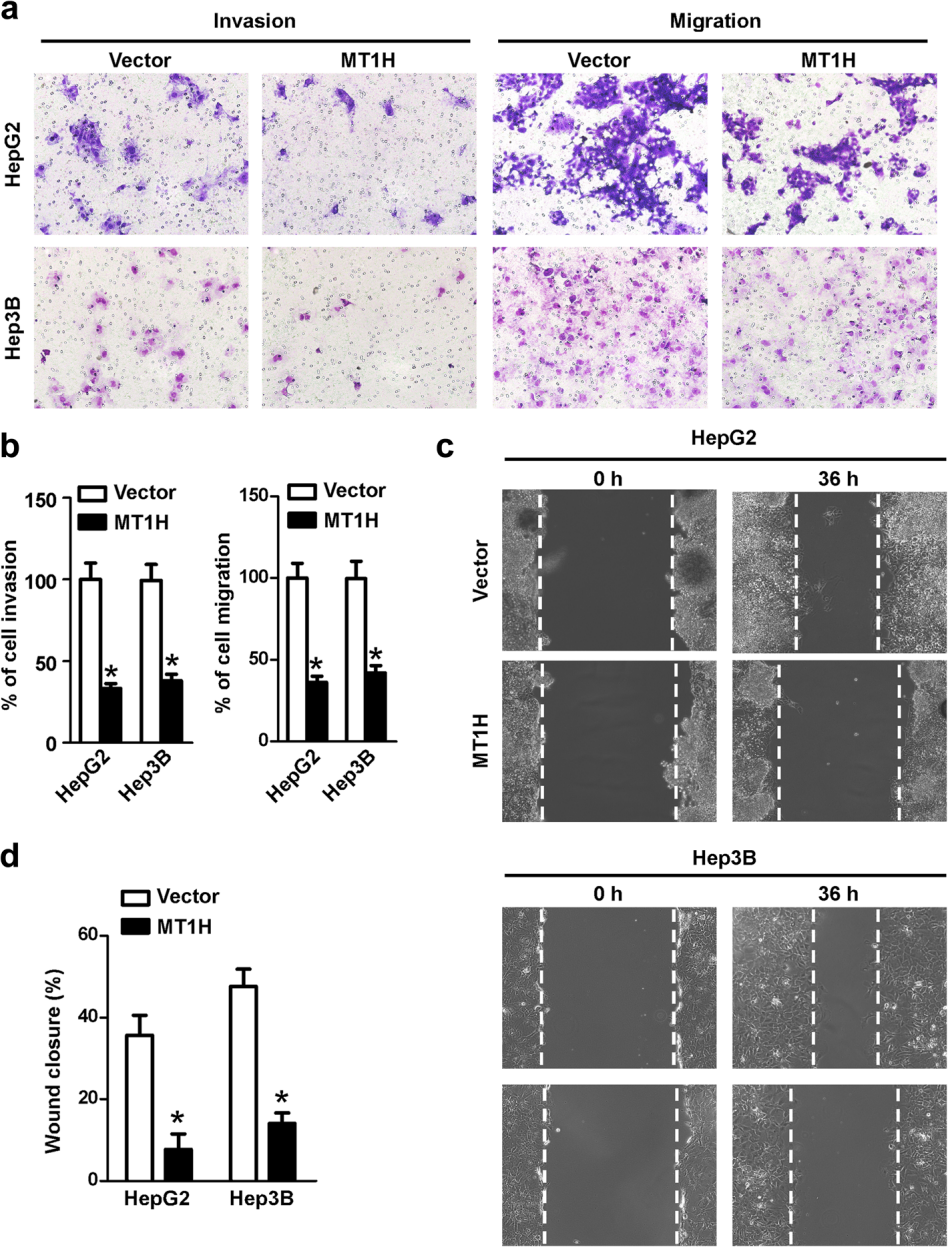
MT1H suppresses the invasion and migration of HCC cells. **a** and **b** (*left*) Matrigel invasion assay showed that invading number of MT1H overexpressing HCC cells was lower than vector-controls. Error bars indicate standard deviation (Student’s *t*-test; *, *P* < 0.05). **a** and **b** (*right*) Transwell migration assay showed that MT1H expression inhibited the migration capability of HCC cells. Error bars indicate standard deviation (Student’s *t*-test; *, *P* < 0.05). **c** and **d** Wound healing assay indicated that MT1H expression inhibited HepG2 and Hep3B cell migration

The effect of MT1H overexpression on invasiveness and migration ability of HepG2 and Hep3B cells was studied. The matrigel-coated transwell assay was performed and the number of MT1H overexpressed cells in the bottom well was significantly reduced compared with the control cells, which indicated that MT1H may suppress the cell invasion (Fig. 3a and b, left). Likewise, the matrigel-uncoated transwell assay showed that ectopic overexpression of MT1H significantly impaired the migration capability of hepatoma cells as compared with the vector control cells (Fig. 3a and b, right). Furthermore, scratch and wound healing assay indicated that enforced expression of MT1H significantly inhibited the migration of HepG2 and Hep3B (Fig. 3c and d). Taken together, these results suggest that MT1H can affect the invasiveness and migration of human HCC cells.

### MT1H suppresses the Wnt/β-catenin signaling via inhibiting nuclear translocation of β-catenin

**Fig. 4:**
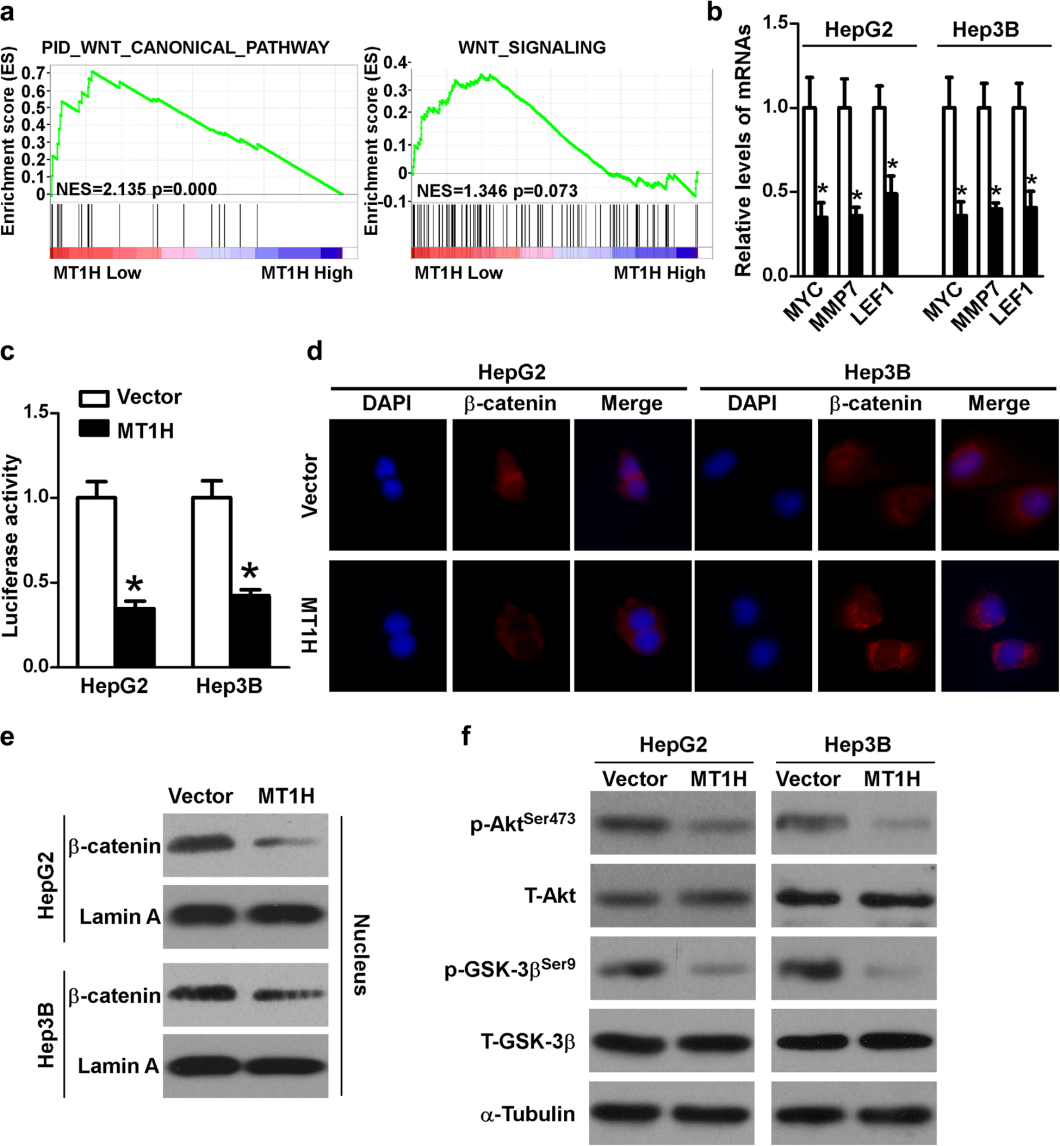
MT1H regulates Wnt/β-catenin signaling in HCC cells. **a** GSEA analysis showed that MT1H might has a regulative role in Wnt/β-catenin signaling in HCC cells. **b** qRT-PCR analysis was used to detect the mRNA levels of *MYC*, *MMP7*, and *LEF1* in indicated cells. Error bars indicate standard deviation (Student’s *t*-test; *, *P* < 0.05). **c** Wnt/β-catenin signaling activity was assessed by TOP/FOP flash dual-luciferase reporter assay in indicated cells. Error bars indicate standard deviation (Student’s *t*-test; *, *P* < 0.05). **d** Immunofluorescence of β-catenin distribution in indicated cells. **e** Western blot analysis of nuclear extraction from indicated cells. **f** Western blot analysis was used to detected the level of p-Akt (Ser 473), total-Akt, p-GSK-3β (Ser 9), and total-GSK-3β in indicated cells. α-Tubulin as a loading control

Next, we sought to explore the driving mechanisms behind the effects of MT1H on HCC cell proliferation, invasion as well as migration. We used LIHC RNAseq2 data generated by the TCGA and sorted the samples into the top and bottom quartiles of *MT1H* expression (high and low *MT1H* expression, respectively). By using TCGA dataset and the GSEA assay, we found that Wnt/β-catenin pathway related genes were significantly enriched in the HCC patients with low *MT1H* expression (Fig. 4a). Moreover, we analyzed whether the association between the *MT1H* expression and Wnt/β-catenin target genes (http://web.stanford.edu/group/nusselab/cgi-bin/wnt/) expression can be found in TCGA data. We categorized the Wnt/β-catenin target genes’ expression into two groups: low expression group (less than the median; *n* = 50) and high expression group (greater than the median; *n* = 50). As shown in Additional file 3: Figure S1a and Additional file 4: Table S3, significantly higher levels of *POU5F1*, *DKK1*, *NOS2*, *MSL1*, *MYCBP*, *NRCAM*, *LEF1*, and *PPARD* transcripts were found in HCC tissues versus adjacent non-tumorous liver tissues. Taken together, these data suggest that MT1H plays a potential role in regulating β-catenin signaling in HCC.

Next, qPCR was performed and the results showed that ectopic overexpression of *MT1H* in HepG2 and Hep3B cells significantly suppressed the levels of *MYC*, *MMP7*, and *LEF1* (Fig. 4b). To further confirm whether MT1H regulates the Wnt/β-catenin signaling, luciferase assay of β-catenin reporter (TOP/FOP) was conducted. As shown in Fig. 4c, MT1H inhibited the Wnt/β-catenin transcriptional activity in HepG2 and Hep3B cells. As expected and shown in Fig. 4d, e, and Additional file 3: Figure S1b, β-catenin nuclear translocation was significantly attenuated in *MT1H* over-expressed HCC cells in comparison with the control cells. Next, we explored the mechanisms of MT1H regulating the β-catenin signaling. We found that the phosphorylation of Akt was significantly inhibited by *MT1H* overexpression (Fig. 4f and Additional file 3: Figure S1c). It has been well known that Akt can lead to phosphorylated inactivation of GSK-3β, and thereby resulting in β-catenin stabilization and nuclear transportation. As expected, the dephosphorylation of Akt reduced phosphorylation of GSK-3β (Fig. 4f and Additional file 3: Figure S1c), suggesting the Akt/GSK-3β axis mediates the modulatory role of MT1H on Wnt/β-catenin signaling in HCC. Collectively, these data indicate that Wnt/β-catenin signaling is a target downstream of MT1H in HCC.

### *MT1H* overexpression attenuates tumorigenicity in nude mice

**Fig. 5:**
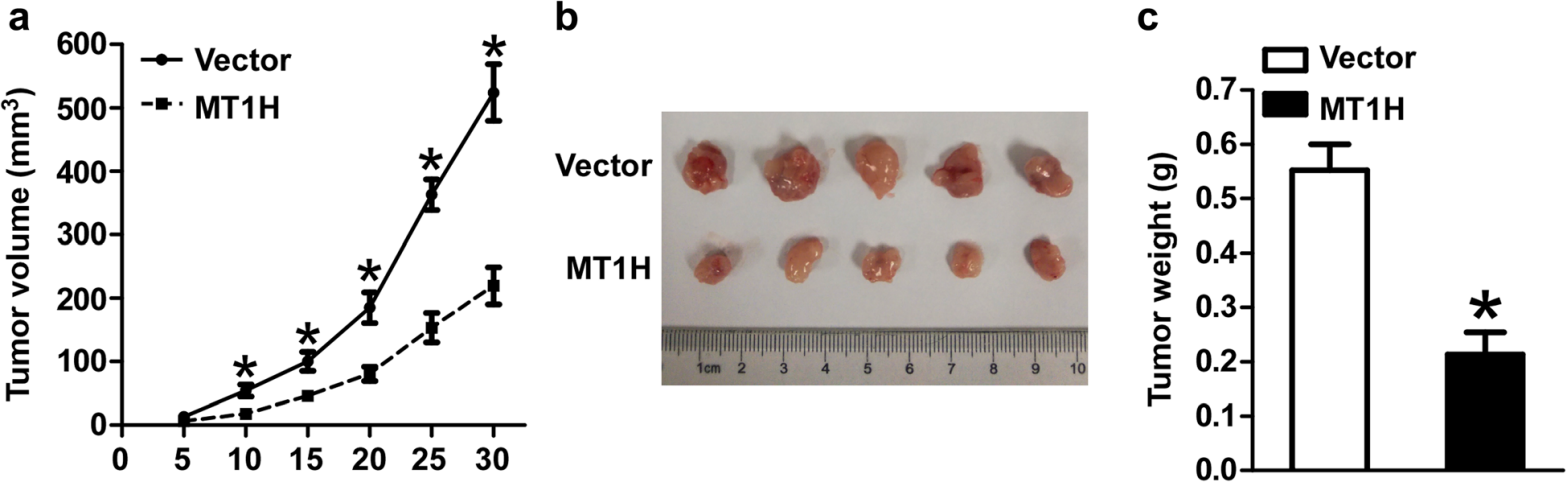
MT1H suppresses tumorigenicity in vivo. **a** Tumor growth curves measured after subcutaneous injection of HepG2-Vector and HepG2-MT1H. The tumor volume was calculated every 5 days. Error bars indicate standard deviation (Student’s t-test; *, *P* < 0.05, *n* = 5). **b** Photographs of dissected tumors from nude mice. **c** Weight of tumors from mice with HepG2-Vector or HepG2-MT1H implantation. Error bars indicate standard deviation (Student’s t-test; *, *P* < 0.05, *n* = 5)

To further assess the effect of *MT1H* overexpression on tumorigenicity in vivo, we subcutaneously injected HepG2-Vector and HepG2-MT1H cells into the nude mice. Intriguingly, as shown in Fig. 5a and b, the tumors formed by HepG2-MT1H cells were significantly smaller than that of control cells. The average tumor weight of the MT1H- transfected cells inoculated mice was significantly decreased as compared with that of control cells, at day 30 (Fig. 5c).

## Discussion

In the present study, we found significantly downregulated *MT1H* in HCC tissues, which is consistent with previous study [14]. Moreover, MT1H plays an anti-proliferative and anti-invasive role in HCC cells. MT1H in HCC can inhibit Wnt/β-catenin signaling, therefore suppresses HCC progression.

MTs are demonstrated to be involved in various physiological and pathological processes, such as protection against oxidative damage, cell proliferation, and apoptosis [11, 28]. Of note, the important roles of MTs in cancer development and progression have been emphasized [29–32]. *MT1H*, one member of *MT-1* genes, has been proved of decreased expression in HCC tissue [14]. Consistently, the results in current study from TCGA database and paired fresh tumor/non-tumor tissues showed significantly decreased *MT1H* expression in HCC tissues. The significant downregulation of *MT1H* in HCC implicates its potential roles in the development and progression of HCC. As expected, our results indicated that ectopic overexpression of *MT1H* may inhibit the growth of HCC cells. Moreover, MT1H involves in the invasiveness of HCC cells.

The mechanisms underlying the downregulation of MTs have been investigated in malignant diseases [12–14, 29, 33–40]. The downregulation of *MT1F* in colon cancer is mainly caused by loss of heterozygosity (LOH) [13]. In addition, a direct relation between p53 and MT-1A and MT-2A was found in epithelial cancer cells [41]. Whether these mechanisms or other mechanism are involved in the downregulation of *MT1H* in HCC need to be further explored. Delineating the precise molecular mechanisms for the altered expression of *MT1H* in HCC will put new insights into the understanding of HCC tumorigenesis.

Given that Wnt/β-catenin pathway plays an essential role in tumorigenesis, together with our bioinformatics analysis implicated that Wnt/β-catenin signaling might involve in the function of MT1H in HCC. It is important to explore the tumor suppressor role of MT1H mediated by Wnt/β-catenin signaling in HCC. We found that MT1H suppressed β-catenin nuclear translocation and transcriptional activity in HCC. Wnt/β-catenin signaling has demonstrated to have important effects on proliferation and invasion of tumor cells. The activated Wnt/β-catenin pathway has been observed in at least one-third HCCs and most of them have mutations in the β-catenin gene [42]. Therefore, it is rational to deduce that tumor suppressive role of MT1H on HCC is attributed, at least partly, to the inhibition of Wnt/β-catenin signaling.

Akt can lead to phosphorylated inactivation of GSK-3β, which in turn results in β-catenin stabilization and nuclear transportation [43]. Of note, Akt signaling has been implicated in mediating the biological functions of MTs [12, 44, 45]. These prompt us to investigate whether MT1H regulates Wnt/β-catenin via Akt/GSK-3β axis. Intriguingly, our results implicate that Akt/GSK-3β axis mediates the modulatory role of MT1H on Wnt/β-catenin signaling. These provide new insights into regulatory networks for dysregulated Akt activation in HCC. Nevertheless, whether MT1H regulates Akt by direct or indirect mechanisms needs to be further explored. Moreover, Han et al. have reported that MT1H interacted with euchromatin histone methyltransferase 1 (EHMT1) and enhanced its methyltransferase activity on histone 3, thereby involved the tumorigenesis of prostate cancer [14]. It remains to be determined whether this mechanism or other mechanisms may also be involved in the role of MT1H in HCC.

## Conclusions

In summary, our data showed that through inhibiting Wnt/β-catenin pathway, MT1H could suppress the proliferation and invasion of HCC cells. MT1H might be a potential new target for HCC therapy. Ongoing studies are to explore the molecular mechanism of downregulated *MT1H* and the interaction between MT1H and Akt/GSK-3β/β-catenin signaling.

## Additional files

Additional file 1: Table S1. Gene sets downloaded from the GSEA. (XLSX 9 kb)
Additional file 2: Table S2. The clinical data of patients analyzed in Fig. 1a. (XLSX 25 kb)
Additional file 3: Figure S1.
**a.** Significantly higher levels of POU5F1, DKK1, NOS2, MSL1, MYCBP, NRCAM, LEF1, and PPARD transcripts in HCC tissues versus adjacent non-tumorous liver tissues. **b and c.** Quantification of indicated protein levels normalized to control using Bio-Rad Quantity One software. Error bars indicate standard deviation (Student’s *t*-test; *, *P* < 0.05). (TIF 379 kb)
Additional file 4: Table S3. The clinical data of patients with low or high MT1H expression. (XLSX 16 kb)

## Abbreviations

APC: Adenomatous polyposis coli
BCLC: Barcelona Clinic Liver Cancer
DKK1: Dickkopf WNT Signaling Pathway Inhibitor 1
DMEM: Dulbecco minimum essential medium
DMSO: Dimethyl sulfoxide
ECL: Enhanced chemiluminescence
EDU: 5-Ethynyl-2′-deoxyuridine
FBS: Fetal bovine serum
GSEA: Gene set enrichment analysis
GSK3beta: Glycogen synthase kinase-3 beta
HCC: Hepatocellular cancer
LEF: Lymphoid-enhancing factor
LOH: Loss of heterozygosity
MMP: Matrix metallopeptidase
mRNA: messenger RNA
MSL1: Male specific lethal 1
MT: Metallothionein
MT1H: Metallothionein 1H
MTT: 3-[4, 5-dimethylthiazol-2-yl]-2, 5-diphenyl-tetrazolium bromide
MYC: V-myc avian myelocytomatosis viral oncogene
MYCBP: MYC binding protein
NOS2: Nitric oxide synthase 2
NRCAM: Neuronal cell adhesion molecule
PCR: Polymerase chain reaction
PI3K: Phosphatidylinositol-3-kinase
POU5F1: POU class 5 homeobox 1
PPARD: Peroxisome proliferator activated receptor delta
PVDF: Polyvinylidene difluoride
RIPA: Radioimmunoprecipitation assay buffer
SDS-PAGE: Sodium dodecyl sulfate-polyacrylamide gels
TBS: Tris-buffered saline
TCF: T-cell factors
TCGA: The Cancer Genome Atlas
