# Supplementary figures and images for: Metallothionein 1H (MT1H) functions as a tumor suppressor in hepatocellular carcinoma through regulating Wnt/β-catenin signaling pathway
Source: BMC Cancer. 2017 Feb 28;17:161. doi: 10.1186/s12885-017-3139-2 (PMC5330125; doi:10.1186/s12885-017-3139-2)

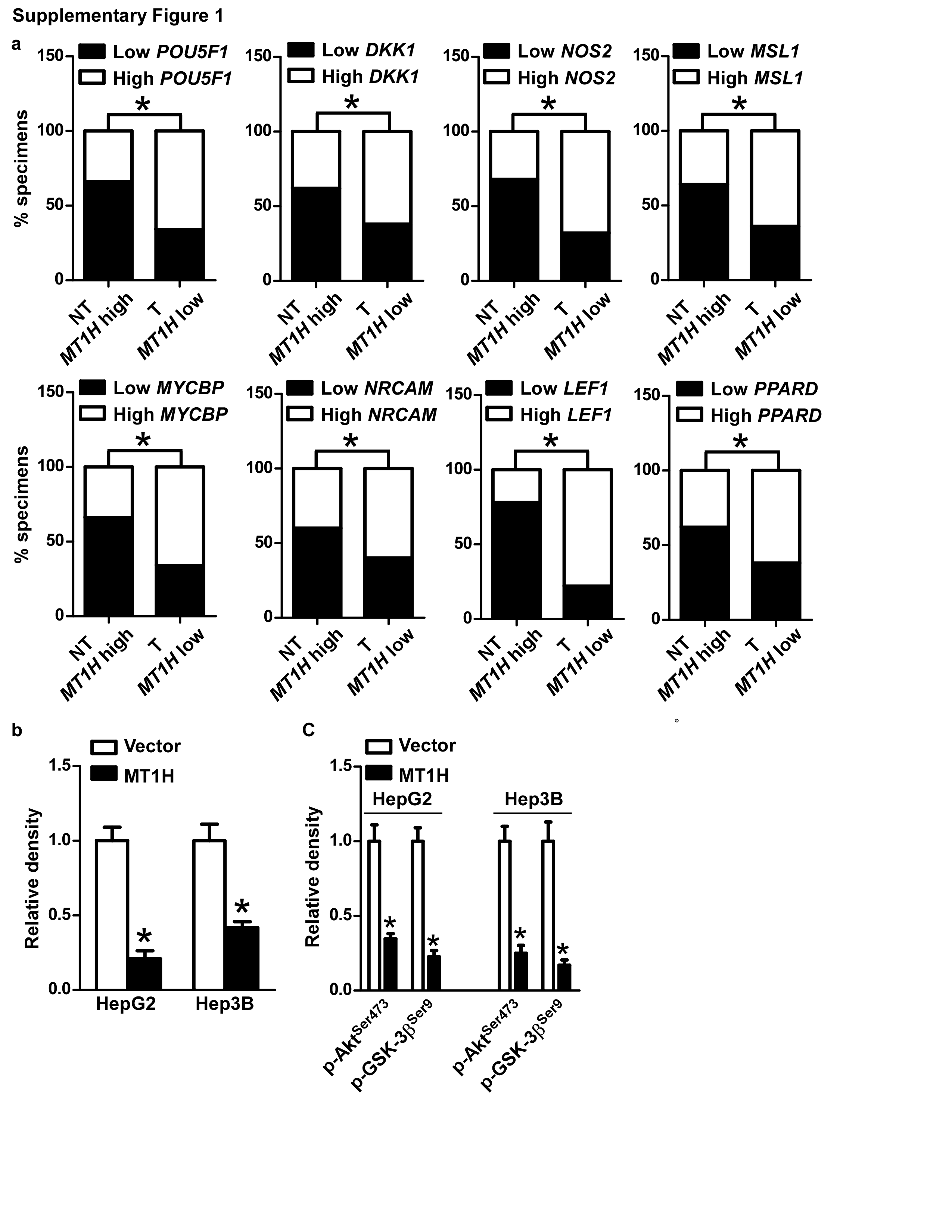

Supplement: Additional file 3: Figure S1. — a. Significantly higher levels of POU5F1, DKK1, NOS2, MSL1, MYCBP, NRCAM, LEF1, and PPARD transcripts in HCC tissues versus adjacent non-tumorous liver tissues. b and c. Quantification of indicated protein levels normalized to control using Bio-Rad Quantity One software. Error bars indicate standard deviation (Student’s t-test; *, P < 0.05). (TIF 379 kb) [file 12885_2017_3139_MOESM3_ESM.tif]
